# Supplementary material for: Non-contact respiration monitoring using impulse radio ultrawideband radar in neonates
Source: R Soc Open Sci. 2019 Jun 5;6(6):190149. doi: 10.1098/rsos.190149 (PMC6599793; doi:10.1098/rsos.190149)
Supplement: Agreement between RRRd and RRIP according to the individual patients [file rsos190149supp1.docx]

Supplementary table 1. Agreement between RR_Rd_ and RR_IP_ according to the individual patients

| Patient | BW on recording (gram) | Age on recording (days) | Recording time (min) | | N | RR | ICC | 95% CI  for ICC | Mean bias | 95% CI  for Mean bias | p-values* | 95% LOA |
| --- | --- | --- | --- | --- | --- | --- | --- | --- | --- | --- | --- | --- |
|  |  |  | Total | Valid |  |  |  |  |  |  |  |  |
| 1 | 3030 | 2 | 130 | 93 | 556 | 42.0±6.8 | 0.71 | 0.67-0.75 | 0.99 | 0.53-1.45 | <0.001 | -9.83-11.81 |
| 2 | 2680 | 6 | 130 | 61 | 361 | 46.0±6.6 | 0.72 | 0.66-0.76 | 0.68 | 0.13-1.23 | 0.017 | -9.8-11.15 |
| 3 | 4060 | 9 | 260 | 169.7 | 396 | 51.6±8.5 | 0.87 | 0.84-0.89 | -0.29 | -0.73-0.15 | 0.199 | -9.12-8.53 |
| 4 | 2930 | 8 | 140 | 116.3 | 696 | 39.8±8.7 | 0.60 | 0.55-0.65 | 4.08 | 3.52-4.65 | <0.001 | -10.82-18.98 |
| 5 | 2820 | 12 | 140 | 98.3 | 588 | 29.6±4.0 | 0.59 | 0.53-0.64 | -0.02 | -0.35-0.31 | 0.909 | -8.04-8.00 |
| 6 | 2940 | 29 | 140 | 63.3 | 377 | 50.1±7.6 | 0.84 | 0.53-0.87 | -0.11 | -0.56-0.34 | 0.629 | -8.89-8.67 |

*Derived from a one-sample t-test

RR_Rd_: respiratory rate measured in the radar; RR_IP_: respiratory rate measured in the impedance pneumography; N: Numbers of data; BW: body weight; RR: respiratory rate; ICC: intraclass correlation coefficient; CI: confidence intervals; LoA: limits of agreement;
